# Seasonal variation in basal and plastic cold tolerance: Adaptation is influenced by both long‐ and short‐term phenotypic plasticity

**DOI:** 10.1002/ece3.3112

**Published:** 2017-06-07

**Authors:** Suegene Noh, Elizabeth R. Everman, Christopher M. Berger, Theodore J. Morgan

**Affiliations:** ^1^ Department of Biology Washington University in St. Louis St. Louis MO USA; ^2^ Division of Biology Kansas State University Manhattan KS USA

**Keywords:** acclimation, daily temperature, developmental plasticity, evolutionary constraint, seasonal temperature

## Abstract

Understanding how thermal selection affects phenotypic distributions across different time scales will allow us to predict the effect of climate change on the fitness of ectotherms. We tested how seasonal temperature variation affects basal levels of cold tolerance and two types of phenotypic plasticity in *Drosophila melanogaster*. Developmental acclimation occurs as developmental stages of an organism are exposed to seasonal changes in temperature and its effect is irreversible, while reversible short‐term acclimation occurs daily in response to diurnal changes in temperature. We collected wild flies from a temperate population across seasons and measured two cold tolerance metrics (chill‐coma recovery and cold stress survival) and their responses to developmental and short‐term acclimation. Chill‐coma recovery responded to seasonal shifts in temperature, and phenotypic plasticity following both short‐term and developmental acclimation improved cold tolerance. This improvement indicated that both types of plasticity are adaptive, and that plasticity can compensate for genetic variation in basal cold tolerance during warmer parts of the season when flies tend to be less cold tolerant. We also observed a significantly stronger trade‐off between basal cold tolerance and short‐term acclimation during warmer months. For the longer‐term developmental acclimation, a trade‐off persisted regardless of season. A relationship between the two types of plasticity may provide additional insight into why some measures of thermal tolerance are more sensitive to seasonal variation than others.

## 1 INTRODUCTION

Climate change is impacting biological systems and affecting average population fitness by shifting phenologies and distributions of species (Kellermann, van Heerwaarden, Sgro, & Hoffmann, 2009; Parmesan & Yohe, 2003; Root et al., 2003). Significant effort to predict the magnitude and severity of the effects of changing temperatures on biodiversity has illustrated that, in order to understand how a species might respond to climate change, we need to determine species dispersal abilities, biotic and abiotic interactions, and adaptive potential (reviewed in Lavergne, Mouquet, Thuiller, & Ronce, 2010). Because increases in climatic variability are predicted to accompany gross climate change, we now expect the impact of climate change to depend more on the variance rather than the mean of temperature change (Jentsch, Jurgen, & Beierkuhnlein, 2007; Vasseur et al., 2014; Wang & Dillon, 2014). This expectation is in large part due to the nonlinear effects of temperature on various aspects of organismal biology (Dell, Pawar, & Savage, 2011). In addition, thermal variation occurs at several time scales making it equally important to understand how population fitness will be affected by shorter‐term fluctuations in temperature due to diurnal and seasonal changes, as well as longer‐term gross fluctuations or extreme events (Marshall & Sinclair, 2012). Understanding how different time scales of selection interact and affect phenotypic and allelic distributions will facilitate better predictions of the effect of climate change on the fitness of populations of many species.

Arthropods comprise a dominant proportion of global biomass and have key roles in many ecosystem processes (Miller, 1993; Wilson, 1987). Adapting to unpredictable change at multiple time scales may be particularly challenging as ectotherm physiology is very sensitive to thermal fluctuations (Deutsch et al., 2008; Foray et al., 2013; Kawecki, 2000). The implications of a changing climate for the survival of insects and other arthropods are often discussed in the context of basal stress tolerance and phenotypic plasticity (Danks, 2005; Sinclair & Roberts, 2005; Sinclair, Vernon, Jaco Klok, & Chown, 2003; Vesala & Hoikkala, 2011). Basal thermal tolerance is a heritable phenotype (Anderson, Hoffmann, & McKechnie, 2005; Ayrinhac et al., 2004; Gerken, Eller, Hahn, & Morgan, 2015; Hallas, Schiffer, & Hoffmann, 2002), and recent work has demonstrated that seasonal fluctuations in basal cold tolerance are linked to fluctuations in allele frequencies in *Drosophila melanogaster*, suggesting that seasonal changes can result in rapid genetic responses to varying environmental stress multiple times per year (Bergland, Behrman, O'Brien, Schmidt, & Petrov, 2014).

Phenotypic plasticity also significantly influences the survival of organisms following thermal stress (Ayrinhac et al., 2004; Deutsch et al., 2008; Geister & Fischer, 2007; Gerken et al., 2015; Kelty, 2007; Kelty & Lee, 2001). Many phenotypes related to thermal stress tolerance are seasonally induced, including pigmentation (Shearer et al., 2016), levels of antifreeze proteins and cryoprotectants (Danks, 2005), and reproductive diapause (Vesala & Hoikkala, 2011; Wallingford, Lee, & Loeb, 2016). Phenotypic plasticity induced through both short‐term acclimation and longer‐term developmental acclimation has been repeatedly shown to increase survival in thermally variable environments in numerous organisms (Basson, Nyamukondiwa, & Terblanche, 2012; Coulson & Bale, 1990; Geister & Fischer, 2007; Hoffmann, Hallas, Dean, & Schiffer, 2003; Lee, Chen, & Denlinger, 1987; Sinclair & Chown, 2006). Short‐term acclimation typically occurs following a brief (minutes to hours) exposure to a nonlethal cool temperature prior to a harsher thermal stress and has ephemeral benefits on survival, wearing off after a few hours (Chen, Denlinger, & Lee, 1987; Czajka & Lee, 1990; Everman, Ledbetter, & Morgan, In Press; Gerken et al., 2015; Kelty & Lee, 2001; Koveos, 2001; Lee et al., 1987; Loeschcke & Sørensen, 2005). While short‐term acclimation can occur in multiple life stages (Rajamohan & Sinclair, 2009), developmental acclimation occurs through exposure of organisms to conditions that alter development and is thus irreversible (Lee et al., 1987; Teets & Denlinger, 2013; Wilson & Franklin, 2002).

Despite this knowledgebase, we do not fully understand how basal and plastic responses to cold stress interact through the seasonal temperature variation characteristic of temperate regions. In particular, a comprehensive understanding of the interaction between basal tolerance and short‐ and long‐term acclimation responses to thermal stress is lacking for species that have complex life cycles (Kingsolver et al., 2011) or produce several generations per year (Bergland et al., 2014). Theory predicts that adaptation to one set of conditions can result in a mismatch between phenotype and environment when conditions shift; however, maintenance of the capacity to respond plastically to shifting environments can reduce this mismatch and facilitate survival of individuals and persistence of populations (Gomez‐Mestre & Jovani, 2013; Kawecki, 2000; Lande, 2014). In addition, it was recently hypothesized that phenotypic plasticity through short‐term and developmental acclimation are evolutionarily linked more closely than previously considered (Beaman, White, & Seebacher, 2016). The capacity for plasticity following developmental acclimation (developmental plasticity), which generally leads to fixed phenotypic effects, should interact with the capacity for acclimation over short timescales (Beaman et al., 2016; Gerken et al., 2015). Short‐term acclimation can reduce the probability that developmental acclimation will result in an irreversible mismatch between phenotype and environment when environmental fluctuation is unpredictable (Beaman et al., 2016). More importantly however, whether acclimation capacity was maintained in Beaman and colleague's model depended on evolutionary cost. It is probable that such costs would also have a seasonal component, for instance with increasing benefits versus costs during more thermally variable times of the year.

We measured the interaction between basal and plastic thermal tolerance as a natural population of *D. melanogaster* responded to seasonal changes in temperatures over multiple years. Specifically, we tested the influence of developmental acclimation on chill‐coma recovery and short‐term acclimation on cold stress survival. Chill‐coma recovery and cold stress survival involve unique genetic mechanisms (Gerken et al., 2015; Morgan & Mackay, 2006), and the different forms of acclimation represent specific temporal scales at which acclimation can occur. Developmental acclimation models seasonal temperature variation experienced during early ontology on the response to thermal stress, while short‐term acclimation models diurnal temperature variation but also has a seasonal context because the magnitude of diurnal temperature variation fluctuates through the season (Colinet & Hoffmann, 2012; Gerken et al., 2015; Kelty & Lee, 2001).

We expected seasonal temperature variation to affect the strength of natural selection on basal cold tolerance for chill‐coma recovery and cold stress survival across the season, but to potentially different extents. In addition, we expected phenotypic plasticity as a result of developmental and short‐term acclimation to improve chill‐coma recovery and cold stress survival. Specifically, we expected less cold tolerant flies from warmer months to still be able to resist cold stress through phenotypic plasticity, despite having experienced weaker natural selection prior to collection. Finally, if a strong constraint exists between basal cold tolerance and plasticity (Gerken et al., 2015; Hoffmann, Sørensen, & Loeschcke, 2003; Kellett, Hoffmann, & Mckechnie, 2005; Nyamukondiwa, Terblanche, Marshall, & Sinclair, 2011), we expect this relationship to be differentially affected by seasonal temperature variation as well (Nylin & Gotthard, 1998). Specifically, because seasonal temperature variation is expected to have a stronger effect on the evolution of chill‐coma recovery, we expect to find a consistent constraint between basal cold tolerance and plasticity in this phenotype. On the other hand, as we expect cold stress survival and acclimation to be less closely related to seasonal variation, the constraint may be less consistently maintained across seasons.

## 2 METHODS

We collected flies through summer and fall of 2012–2015 from two commercial orchards in Topeka, KS (39.09 N, −95.59W and 39.20 N, −95.74W) that are 11 miles apart. In 2012 and 2013, we collected flies at three different times (July, September, October), in 2014 at five different times (June, July, August, September, November), and in 2015 at three different times (July, September, October). We attracted flies by placing fermented banana bait traps near or hanging onto apple trees for 2–3 days. These traps were 1 L plastic bottles with a single curved opening approximately 3 inches wide made on one side. We did not distinguish between flies from each orchard, as they were geographically close and environmentally similar (growing the same types of fruits). For each collection, we placed four traps at each orchard. For each collection time, we isolated *D. melanogaster* or *D. simulans* females into individual vials with standard cornmeal‐molasses food and allowed them to lay eggs. After 1 week in a vial, we moved these founder females into another food vial once more. We identified to species the isofemale lines we established in this way by checking the genital morphology of male offspring. We retained only *D. melanogaster*, and maintained and inbred isofemale lines or mass population cages established from ten isofemale lines each, depending on the experiment. More details are given below.

### 2.1 Chill‐coma recovery and developmental acclimation

We measured chill‐coma recovery in flies collected from 2012–2014. Isofemale lines established in 2012 and 2013 were maintained for 5–8 generations at 25°C prior to chill‐coma recovery phenotyping. Flies from 2014 isofemale lines were reestablished from mass population cages and maintained for 2 generations at 25°C prior to phenotyping. To determine the plastic effect of developmental acclimation on chill‐coma recovery, we reared the isofemale lines from 2012 and 2014 at 18°C for an additional 3–5 generations and phenotyped them after developmental acclimation.

We used an automated phenotyping technique to score up to 200 flies at a time for chill‐coma recovery as described in (Crawford, 2013). Briefly, we placed a gridded phenotyping stage in an incubator set to 25°C. Above the stage, we positioned a digital SLR camera (Canon EOS Rebel T3) that photographed all the grids within its view. We used camera software (DSLR Remote Pro for Windows) to automatically take photos at 60‐s intervals. We replicated each assay twice per sex, per line and developmental environment. Experimental flies were sexed using light CO_2_ anesthesia, and 8–11 single sex flies were placed in empty vials. Chill‐coma was measured as in Morgan and Mackay (2006); specifically, we set 20 vials into a refrigerator set to 0°C for 3 hr. After 3 hr, vials were removed the rack from the refrigerator and emptied each vial into a cell of the gridded stage as quickly as possible. We positioned individuals within the grid so that they were on their backs and sufficiently spaced so that none were touching each other. This usually took 3–4 min. Resistant populations of *D. melanogaster* do not recover from a 3 hr exposure to 0°C before 6–7 min (Gerken, Mackay, & Morgan, 2016). Thus, we took photos every minute from 5 min postremoval from the refrigerator to 40 min postremoval. After 40 min, flies were removed from the staged incubator.

We used custom code to score positions of flies at each minute interval using a fiji (ImageJ) script that directed the tool ParticleAnalyzer to report locations of flies. We scored the waking time of each fly by comparing the locations of flies from minute to minute. Any fly that shifted position between camera frames was considered awake. Any flies that had not moved by the end of phenotyping were given “41” min for waking time because the vast majority of flies that were still immobile at this time would move once nudged with the hand vacuum used to clear the phenotyping stage.

### 2.2 Cold stress survival and short‐term acclimation

We mass‐reared flies collected in 2014 and 2015 for two generations at 25°C prior to cold stress survival phenotyping. We established four to six mass‐reared outbred population cages for each collection time from approximately 10 isofemale lines each to circumvent inbreeding effects in this harsher fitness assay.

To measure cold stress survival, we obtained flies from mass population cages 2 days posteclosion. From each population bottle, we sorted flies by sex on a CO_2_ stage into vials containing 20 individuals apiece. Flies were allowed to recover and mature for 5 days prior to phenotyping. We measured cold stress survival by exposing one set of experimental flies to −6°C for 1 hr (nonacclimation treatment). We measured short‐term acclimation through rapid cold‐hardening by exposing a second set of the experimental flies first to 4°C for 2 hr, immediately followed by exposure to −6°C for 1 hr (acclimation treatment). Cold stress and acclimation temperatures were chosen following Gerken et al. (2015). We recorded survival per vial after a 24 hr recovery period at 25°C with access to food. We replicated each assay twice per sex, per bottle for each acclimation treatment.

### 2.3 Seasonal temperature variation and statistical analysis

To compare the effect of seasonal environment across years, we compiled data from degreedays.net regarding the cooling and heating degree days for the 14 days (the approximate length of a single generation for *D. melanogaster* in the lab at 25°C) leading up to and including each collection date, from Topeka Billard Municipal Airport (KTOP: 39.07 N, 95.62 W, an average distance of 6 miles from the collection sites). Heating degree days are the cumulative degrees air temperatures that fell below a reference temperature (and thus required heating to maintain that temperature), while cooling degree days are the cumulative degrees air temperatures that were above a reference temperature (and required cooling). We selected 25°C and 18°C for the cooling and heating degree day reference temperatures because these were the rearing temperatures used to examine the effect of developmental temperature. We found that our collection dates over the 3 years were relatively evenly sampled and less skewed across the range of cooling degree days with a reference temperature of 18°C (CDD18 (skewness = 0.13)) compared to the alternatives (HDD25 (skewness = 1.19), HDD18 (skewness = 1.59), CDD25 (skewness = 1.15)). From here on, we use cumulative heat exposure above 18°C (CDD18) as a proxy for the seasonal weather experienced by the isofemale line founders. We imported these compiled data into R v.3.2.1 for statistical analysis (R Core Team, 2015).

For chill‐coma recovery, we only included lines from which we were able to get data from at least 39 individuals total (both female and male). In 2012, we collected recovery time data from a total of 101 lines of flies (July—33 lines, September—34 lines, October—34 lines). In 2013, we collected data from 90 lines of flies (July—30 lines, September—30 lines, October—30 lines). In 2014, we collected data from 100 lines of flies (June—30 lines, July—30 lines, August—21 lines, September—19 lines). Exploratory examination of our data suggested that chill‐coma recovery waking times were not normally distributed but instead fit a quasipoisson pattern, with mean and variance showing a positive linear relationship. Compared to data with a Poisson distribution, with quasipoisson data the variance increases at a rate above 1 as the mean increases. Therefore, we chose to analyze our data with penalized quasilikelihood generalized linear mixed models fit with a quasipoisson error distribution and its accompanying log link function using the R library MASS v.7.3‐44 (Venables & Ripley, 2002). For cold stress survival, again we only included cages from which we were able to get data from at least 39 individuals total (both female and male) and the median number of flies that survived the cold survival assay across replicates was at least one individual. In 2014, we collected survival data from 30 cages of flies (July—6 cages, August—4 cages, September—6 cages, October—6 cages, November—8 cages). In 2015, we collected data from 26 cages of flies (June—6 cages, July—8 cages, August—6 cages, September—6 cages). Because the response variable for cold stress survival is binary (alive, dead), we analyzed these data using a generalized linear mixed model fit with a binomial error distribution and its accompanying log link function using the R library lme4 v.1.1‐10 (Bates, Mæchler, Bolker, & Walker, 2015). From hereon, we will refer to both types of models simply as mixed effects models.

We used the flies from the 2012–2014 25°C chill‐coma recovery experiments and 2014–2015 nonacclimated cold stress survival experiments to test the hypotheses: (i) seasonal temperature variation affects basal cold tolerance through natural selection, and (ii) developmental and short‐term acclimation compensates for the genetic differences in cold tolerance. For the chill‐coma recovery data, we fit a mixed model to waking time. We used CDD18 and developmental temperature and their interaction as fixed factors and sex, nested in lines, nested in collection years as random factors. For the cold stress survival data, we fit a mixed model to the binomial variable of flies alive versus dead. We used CDD18 and acclimation treatment as fixed factors and sex, nested in cages, nested in collection years as random factors. We were unable to test the interaction effect between CDD18 and acclimation because these models failed to converge. All models were fit with the continuous variable CDD18, but to make residual plots easier to visualize, we created a categorical variable from CDD18 (“low”, “mid”, and “high”) by simply dividing each range of CDD18 into thirds. For both chill‐coma recovery and cold stress survival, the effect of seasonal temperature on basal tolerance (hypothesis i) was tested by assessing the effect of CDD18 on each response variable, and the effect of developmental and short‐term acclimation (hypothesis ii) was tested by assessing the effect of developmental temperature or acclimation treatment.

Finally, we used flies from the 2012 and 2014 25°C and 18°C chill‐coma recovery experiments and 2014–2015 short‐term acclimation experiments to test the hypothesis: seasonal temperature variation affects the trade‐off between basal cold tolerance and plasticity. We estimated developmental plasticity by taking the difference between recovery times in the two development treatments (development at 25°C vs. 18°C) for each line. We calculated an acclimation score by taking the difference between survival in the two cold‐hardening treatments (acclimation and nonacclimation) for each population cage. We looked at the correlation between each type of cold tolerance metric and its corresponding plasticity metric within levels of CDD18. We used the same CDD18 categories as described above. We used linear models to test whether the relationships between basal tolerance and plasticity were significantly different by CDD18 category.

## 3 RESULTS

### 3.1 Seasonal variation in basal cold tolerance and plasticity

**Figure 1:**
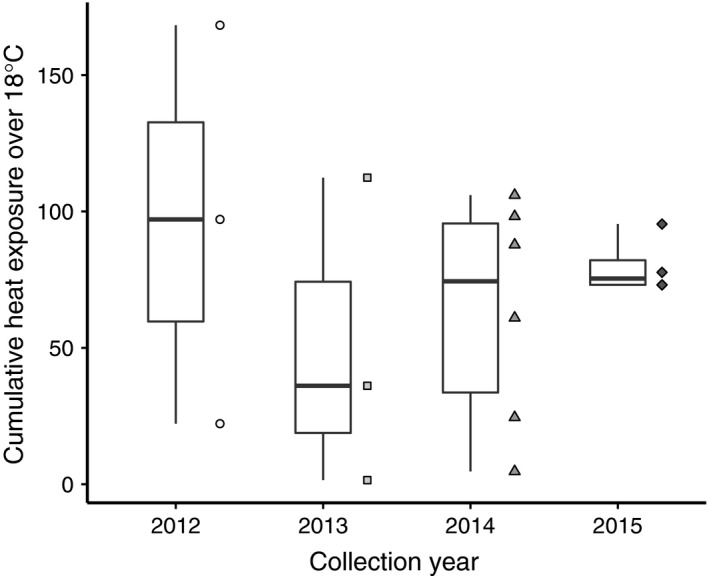
Cooling degree days above 18°C (CDD18) for the collection dates of isofemale line founders used in the experiments. Degree days are cumulative for the 14 days prior to and including the collection date and were obtained from the nearest weather station located at Topeka Municipal Airport. Symbols indicate the average CDD18 for each collection date, each year

We used cooling degree days with an 18°C reference (CDD18) as our proxy for the seasonal temperature experienced by founder females of the isofemale lines we tested in our experiment. Across the four collection years, we observed a wide range in thermal variation from an average of 28.7°C each day over the 2 weeks preceding collection in the warmest month (July, 2012) to approximately 18°C over the 2 weeks preceding collection in the coolest months (September, 2013 and November, 2014; Figure 1). This natural seasonal temperature variation affected the basal cold tolerance for chill‐coma recovery but not cold stress survival in flies, while plasticity in the form of developmental and short‐term acclimation improved cold tolerance across all flies and compensated for any differences among flies collected during different seasons.

#### 3.1.1 Chill‐coma recovery and developmental acclimation

**Figure 2:**
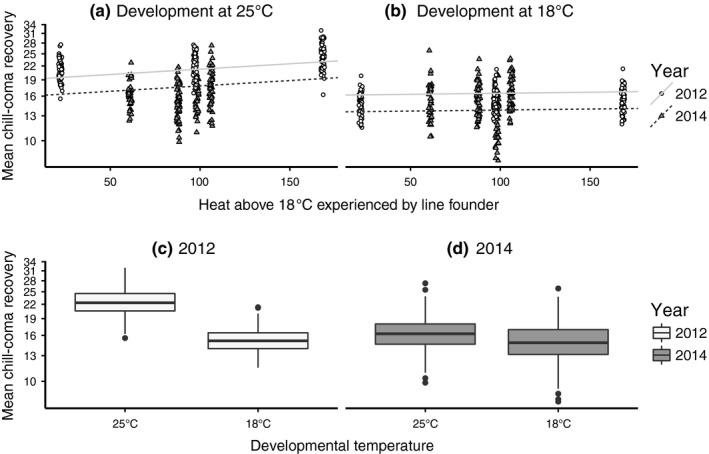
(a‐b) Mixed effects model fit to chill‐coma recovery times of flies founded from females collected at different times of the season and reared in the lab at 25°C and subsequently at 18°C to measure the effect of developmental acclimation. Linear models shown incorporate both fixed and random effects coefficients obtained from the mixed effects model. Each point represents one replicate of one sex of one isofemale line. (c‐d) The average effect of developmental acclimation shown by year. Note that the *y*‐axis is in log scale because Quasipoisson errors are log linked

**Figure A1:**
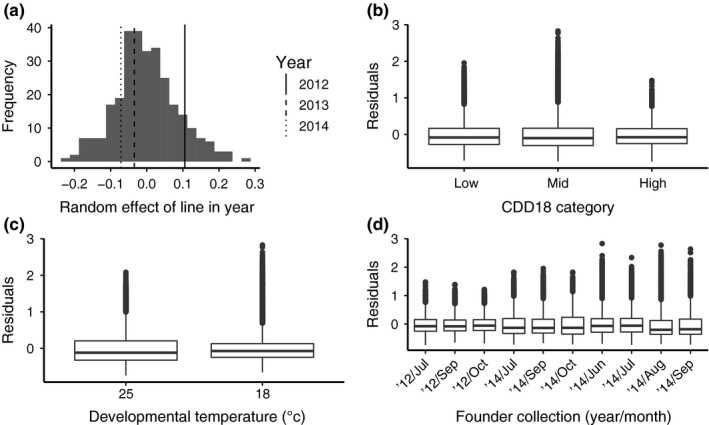
(a) Distribution of random effects fit to chill‐coma recovery time of flies reared at 25°C and then 18°C. The histogram is of the random effect intercepts fit to isofemale lines nested in year. Vertical lines are random effect intercepts fit to collection year. (b–d) Residuals of the mixed effects model

Flies that experienced more cumulative heat exposure were slower to recover from chill‐coma (β_CDD18_ = 0.001 ± <0.001, *t* = 7.09, *p* < .001) (Figure 2a). This effect was significant despite the “common garden” rearing and maintenance of flies at 25°C in the lab. To determine the effect of developmental acclimation, we moved these flies to a constant 18°C and tested chill‐coma recovery once more. This switch in developmental temperature from 25°C to 18°C shortened recovery times (β_Development_ = −0.16 ± 0.01, *t* = −14.96, *p* < .001; Figure 2b–d), and interacted with CDD18 to reduce the effect of CDD18 on recovery time (β_CDD18:Development_ = −0.001 ± <0.001, *t* = −8.83, *p* = <.001; Figure 2b). Males generally recovered faster, and this difference was accounted for as a random intercept in our model. The variation between the sexes (*SD* = 0.07), among lines established from females collected at the same time (*SD* = 0.10), and among collection years (*SD* = 0.08) were similar. Visual examination of the random effects coefficients and boxplots of the residuals did not show extreme outliers or potential issue due to variance heterogeneity (Figure A1).

#### 3.1.2 Cold stress survival and short‐term acclimation

**Figure 3:**
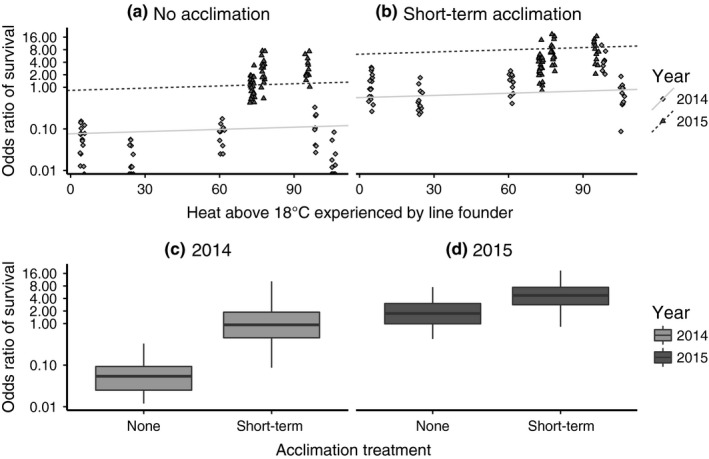
(a‐b) Mixed effects model fit to cold stress survival (alive vs. dead) from females collected at different times of the season and reared in the lab at 25°C. For the short‐term acclimation treatment, flies were exposed to a milder cold temperature (4°C) prior to the survival assay at −6°C. Linear models shown incorporate both fixed and random effects coefficients obtained from the mixed effects model. Each point represents one replicate of one sex of one population cage. (c‐d) The average effect of short‐term acclimation shown by year. Note that the *y*‐axis is in log scale because binomial errors are log linked

**Figure A2:**
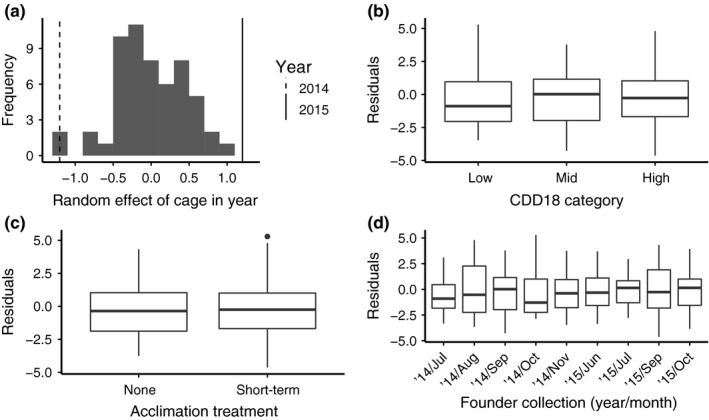
(a) Distribution of random effects fit to cold stress survival of flies exposed to cold stress at −6°C with and without short‐term acclimation at 4°C. The histogram is of the random effect intercepts fit to population cages nested in year. Vertical lines are random effect intercepts fit to collection year. (b–d) Residuals of the mixed effects model

Survival following short‐term cold stress in flies was not significantly influenced by cumulative heat exposure (odds ratio β_CDD18_ = 1.00, 95% CI (0.990–01.01), *z* = 1.40, *p* = .16) (Figure 3a). To determine the effect of short‐term acclimation, we exposed these flies to a 4°C rapid cold‐hardening (RCH) treatment and tested cold stress survival once more. This treatment significantly increased cold stress survival (odds ratio β_Treatment_ = 7.40, 95% CI (6.76–8.09), *z* = 43.98, *p* < .001) (Figure 3b–d). Males generally survived better than females (*SD* = 0.41), and this variation was similar in magnitude to the variation among population cages established from females collected at the same time (*SD* = 0.56). However, the variation among collection years was much larger (*SD* = 1.22) and as a result, there was more variation between years than within (Figure A2a). Visual examination of the boxplots of the residuals distributed across fixed and random effects did not show extreme outliers or potential issues due to variance heterogeneity (Figure A2b–d).

### 3.2 Evidence of seasonal variation in the trade‐off between basal cold tolerance and acclimation

**Figure 4:**
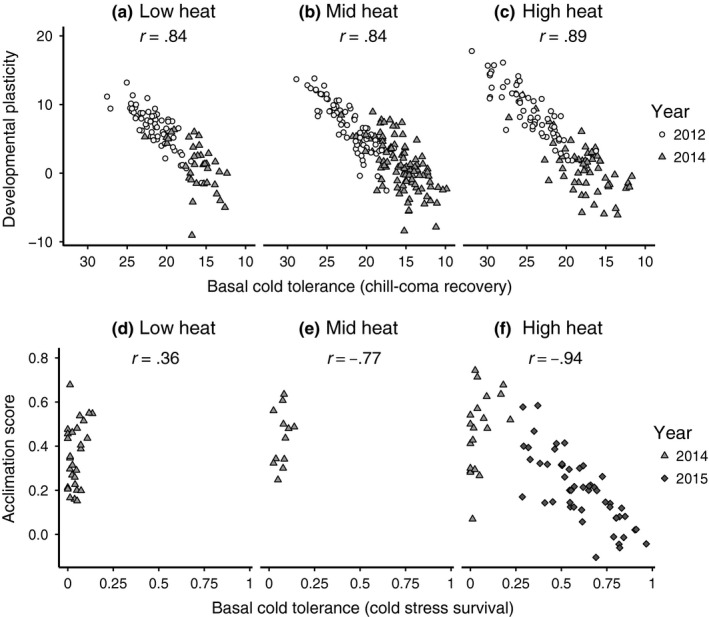
(a–c) Basal chill‐coma recovery and its relationship with developmental plasticity across the range of cumulative heat exposure experienced by founder females of isofemale lines (individual points represent one sex of one line). (d–f) Basal cold stress survival and its relationship with short‐term acclimation across the range of seasonal temperature variation experienced by founder females of population cages (individual points represent one sex of one cage). Correlations between the relevant metrics are shown individually for each panel

We found that for both chill‐coma recovery and cold stress survival, the basal cold tolerances and their respective plasticity measures showed significant relationships (Figure 4). Flies with higher basal cold tolerance will have a shorter chill‐coma recovery time (note that the *X*‐axis in Figure 4a–c is flipped in orientation because of this). Thus, the association between basal cold tolerance and developmental plasticity is negative, and flies with higher basal cold tolerance for chill‐coma recovery showed the least amount of developmental plasticity. This association was present regardless of the level of cumulative heat exposure, so that whether it was low or high, basal cold tolerance for chill‐coma recovery and developmental plasticity always showed a similar degree of association (CDD18_Level_:CCR_25_, F_2_ = 0.75, *p* = .48; Figure 4a–c). Cold stress survival also showed an overall negative relationship with short‐term acclimation plasticity, and flies with higher basal cold tolerance for cold stress survival showed the least benefit from short‐term acclimation. In contrast to chill‐coma recovery, this association was strongest in flies with a higher level of cumulative heat exposure than in flies with less cumulative heat exposure (CDD18_Level_:CSS_NON_, F_2_ = 3.75, *p* = .03; Figure 4d–f).

## 4 DISCUSSION

Temperature fluctuations are effective sources of natural selection for small ectothermic organisms with short generation times (Bergland et al., 2014; Kelty & Lee, 2001; MacMillan & Sinclair, 2011; Vesala & Hoikkala, 2011). Over the 4 years that we sampled a natural midcontinent population of *D. melanogaster*, the seasonal temperature variation experienced by founding females decreased each successive year (Figure 1). Despite the more consistent summer and fall temperatures in progressive years, we detected a significant effect of cumulative heat exposure in flies that were reared at 25°C under common garden conditions and tested for chill‐coma recovery time (Figure 2a). Flies collected during warmer months typically took longer to recover from chill‐coma, suggesting that natural populations have decreased cold tolerance during this part of the year. Cold tolerance increased as cumulative heat exposure decreased, indicating that seasonal change in temperatures from summer to fall across the 3 years influenced cold tolerance in the expected direction as measured by chill‐coma recovery. The significant effect of cumulative heat exposure on chill‐coma recovery in flies reared at 25°C is a signal of genetic change as this natural population adapts to temperature variation throughout the seasons each year. Cyclical changes in selection pressure have repeatedly been shown to influence fitness and life history phenotypes (Behrman, Watson, O'Brien, Heschel, & Schmidt, 2015; Bergland et al., 2014; Betini, Griswold, Prodan, & Norris, 2014). Over short stretches of time, these cyclical selection pressures can cause high‐frequency alleles that were beneficial earlier in the season to become less frequent when they are less beneficial. Bergland et al. (2014) found evidence to support this pattern of allele frequency fluctuation and further linked specific fluctuating loci to chill‐coma recovery. Behrman et al. (2015) also observed oscillating cold tolerance phenotypes in both *D. simulans* and *D. melanogaster*.

Cold tolerance assessed through exposure to short‐term cold stress did not recapitulate this pattern (Figure 3a). Flies collected during warmer months and during colder months did not differ in cold stress survival. It is important to note that the variance between collection years in cold stress survival was larger than the variance within each year for this metric. Larger differences in environmental conditions experienced by founding females may be necessary to elicit a change in this measure of cold stress survival. Thus, it is quite possible that we were unable to detect a significant effect of season on cold stress survival due to the relatively small degree of temperature fluctuation across our collection dates during 2014 and 2015, as opposed to those in 2012 and 2013 (Figure 1). This is especially true for females collected in 2015, where temperatures during the 2 weeks preceding each collection period were similar across a 4‐month period of time (Figure 1). Our decision to use outbred population cages to assess cold stress survival may have also influenced the difference in results compared to chill‐coma recovery. However, if this were the case, the effect of having fewer population cages versus more isofemale lines is likely to make any differences easier to detect by decreasing the variation among genotypes. Therefore, we draw similar conclusions to prior studies that have reported a lack of seasonal variation in extreme cold stress tolerance (Hoffmann & Watson, 1993) and suggest that cold stress survival may not respond to seasonal temperature variation. Given that tolerance to short‐term, severe thermal stress is expected to be important for surviving daily fluctuations in temperature (Hoffmann & Watson, 1993; Kelty & Lee, 2001; Lee et al., 1987), our results may not be surprising.

In addition to the positive effect of seasonal temperature variation on cold tolerance at least for chill‐coma recovery, both short‐term acclimation and longer‐term developmental acclimation improved cold tolerance (Figures 2c–d and Figure 3c–d). Thus, both forms of phenotypic plasticity are adaptive because they allow warmer season flies to effectively recover the cold tolerance of cooler season flies that are selected by seasonal temperature variation to be more basally cold tolerant. As with similar lab‐based tests of adaptation, we cannot rule out the potential influence of lab adaptation during common garden rearing on our insects, for instance during the 3–5 generations of development at 18°C. However, the large degree of plasticity we observed in our population of flies and consistency of patterns across years is in line with previous reports of adaptive plasticity found for cold tolerance phenotypes, including chill‐coma recovery (Ayrinhac et al., 2004; Rako & Hoffmann, 2006) and cold stress survival (Gerken et al., 2015; Lee et al., 1987).

Finally, basal cold tolerance and adaptive plasticity for both types of acclimation showed a characteristic trade‐off pattern, in which the capacity for phenotypic plasticity was greater for less basally cold tolerant lines or cages and vice versa (Figure 4). Trade‐offs between basal cold tolerance and plasticity have been reported before (Gerken et al., 2015; Hoffmann, Sørensen, et al., 2003; Kellett et al., 2005; Nyamukondiwa et al., 2011). While the relationship is naturally biased toward a negative relationship, the slope describing this relationship within a single population over time provides insight into the dynamics of such trade‐offs (Sørensen, Kristensen, & Overgaard, 2016). A previously untested but important aspect of the trade‐off between basal tolerance and adaptive plasticity is whether the relationship constrains how individual organisms can respond to seasonal variation. Our results suggest that when a consistent trade‐off is maintained across seasons, it can help organisms adapt to seasonal changes. For chill‐coma recovery, the tight and nonfluctuating relationship between basal cold tolerance and developmental plasticity indicates that these insects will recover from chill‐coma fairly well regardless of season (Figure 4a–c). During warmer months, the same can be said for cold stress survival. However, during colder months, all population cages had equally poor basal cold tolerance even though they retained a fairly large range of acclimation capacities (Figure 4d–f). To understand this difference, further research is needed to determine whether natural selection maintains this consistent trade‐off between basal tolerance and plasticity due to the seasonal nature of chill‐coma recovery or that of developmental plasticity, as basal cold tolerance for chill‐coma recovery but not cold stress survival responded to seasonal temperature variation.

The difference in the dynamics of the trade‐off in chill‐coma recovery versus cold stress survival may also reflect a constraint that exists between the two types of phenotypic plasticity. Developmental acclimation results in an irreversible type of plasticity while short‐term acclimation is generally reversible (Kelty & Lee, 2001; Koveos, 2001). Maintaining both types of plasticity can be particularly advantageous in species that have short generation times and reproduce multiple times a year, though the relative capacity of each type of acclimation would depend on the evolutionary cost of maintaining short‐term acclimation capacity in particular (Beaman et al., 2016). We are unable to test whether the difference in trade‐offs we observed is the effect of a stronger constraint in the relationship between developmental and short‐term acclimation during different seasons because we tested the effect of acclimation on two different cold tolerance phenotypes. As a result, our ability to interpret the differences in seasonal patterns in short‐term and developmental acclimation capacity in our study is limited. However, the potential for this type of multivariate relationship between basal tolerance and phenotypic plasticity may provide additional insight into why some measures of thermal tolerance are more sensitive to seasonal variation than others, and could extend to broader spatial scales as well.

We expect natural thermal environments to fluctuate, and fluctuations that occur within the thermal performance range of an ectotherm typically increase its fitness (Colinet, Chertemps, Boulogne, & Siaussat, 2015). We began our discussion by noting how much less variable each successive year from 2012 to 2015 was in terms of the metrics of thermal variation we used (Figure 1). Year by year, the basal tolerances of flies increased for chill‐coma recovery from 2012 through 2014 and increased for cold stress survival from 2014 to 2015. If the reduced temperature variability and increase in basal tolerance over the last few years led to reduced allelic variation or capacity for plasticity, extended atypical weather, such as an extreme cold spell during a warmer season, would present a serious challenge for this natural population. While plasticity has positive affects on cold tolerance within the range of thermal stresses we tested in this natural population of flies, the predictability and magnitude of climatic changes going forward is certain to influence the persistence of this population.

## CONFLICT OF INTEREST

None declared.
